# Design, implementation and evaluation of a national campaign to distribute nine million free LLINs to children under five years of age in Tanzania

**DOI:** 10.1186/1475-2875-10-73

**Published:** 2011-03-31

**Authors:** Kimberly Bonner, Alex Mwita, Peter D McElroy, Susan Omari, Ally Mzava, Christian Lengeler, Naomi Kaspar, Rose Nathan, Joyce Ngegba, Romanus Mtung'e, Nick Brown

**Affiliations:** 1 National Malaria Control Programme, Ministry of Health and Social Welfare, PO Box 9083, Dar es Salaam, Tanzania; 2 President's Malaria Initiative, American Embassy PO Box 9123, Dar es Salaam, Tanzania; 3 ITN Cell, Swiss Tropical and Public Health Institute, PO Box 3430, Dar es Salaam Tanzania; 4 Health Interventions Unit, Swiss Tropical and Public Health Institute, PO Box 4002, Basel, Switzerland; 5 University of Basel, Basel, Switzerland; 6 Ifakara Health Institute, PO Box 78373, Dar es Salaam, Tanzania; 7 World Vision Tanzania, PO Box 6399, Dar es Salaam, Tanzania; 8 Population Services International, PO Box 33500, Dar es Salaam, Tanzania

## Abstract

**Background:**

After a national voucher scheme in 2004 provided pregnant women and infants with highly subsidized insecticide-treated nets (ITNs), use among children under five years (U5s) in mainland Tanzania increased from 16% in 2004 to 26.2% in 2007. In 2008, the Ministry of Health and Social Welfare planned a catch-up campaign to rapidly and equitably deliver a free long-lasting insecticidal net (LLIN) to every child under five years in Tanzania.

**Methods:**

The ITN Cell, a unit within the National Malaria Control Programme (NMCP), coordinated the campaign on behalf of the Ministry of Health and Social Welfare. Government contractors trained and facilitated local government officials to supervise village-level volunteers on a registration of all U5s and the distribution and issuing of LLINs. The registration results formed the basis for the LLIN order and delivery to village level. Caregivers brought their registration coupons to village issuing posts during a three-day period where they received LLINs for their U5s. Household surveys in five districts assessed ITN ownership and use immediately after the campaign.

**Results:**

Nine donors contributed to the national campaign that purchased and distributed 9.0 million LLINs at an average cost of $7.07 per LLIN, including all campaign-associated activities. The campaign covered all eight zones of mainland Tanzania, the first region being covered separately during an integrated measles immunization/malaria LLIN distribution in August 2008, and was implemented one zone at a time from March 2009 until May 2010. ITN ownership at household level increased from Tanzania's 2008 national average of 45.7% to 63.4%, with significant regional variations. ITN use among U5s increased from 28.8% to 64.1%, a 2.2-fold increase, with increases ranging from 22.1-38.3% percentage points in different regions.

**Conclusion:**

A national-level LLIN distribution strategy that fully engaged local government authorities helped avoid additional burden on the healthcare system. Distribution costs per net were comparable to other public health interventions. Particularly among rural residents, ITN ownership and use increased significantly for the intended beneficiaries. The upcoming universal LLIN distribution and further behaviour change communication will further improve ITN ownership and use in 2010-2011.

## Background

Many African countries are in the midst of unprecedented efforts to rapidly scale-up coverage of malaria interventions, but considerable work remains. As recently as 2007-08, prevalence of *Plasmodium falciparum *parasitaemia exceeded 40% in some regions of Tanzania [1]. Through committed political leadership and support from multilateral and bilateral donors, mainland Tanzania now implements all four malaria control strategies recommended by the Roll Back Malaria (RBM) Partnership [2,3]. The strategies include insecticide-treated bed nets (ITNs) (since 2004), intermittent preventive treatment for pregnant women (since 2006), artemisinin-based combination therapy as first-line treatment (since 2007), and indoor residual spraying in selected areas (since 2008) [4]. However, scale-up of these interventions has not been uniformly achieved across all geographic areas of Tanzania, and disparity exists across urban/rural and wealth strata [5-9].

The National Insecticide-Treated Nets Programme (NATNETS) under the National Malaria Control Programme (NMCP) of the Ministry of Health and Social Welfare (MoHSW) is a multi-donor, multi-partner initiative to promote the national use of ITNs by making nets affordable, accessible, and acceptable. In 2004, NMCP initiated the Tanzania National Voucher Scheme (TNVS), a distribution mechanism supported by a Round 1 grant from the Global Fund to Fight AIDS, TB, and Malaria (GFATM) for delivering subsidized polyester nets bundled with insecticide treatment kits to pregnant women at antenatal visits [5]. Through support from the US President's Malaria Initiative in 2006, the TNVS added an infant voucher delivered at time of routine measles immunization. Between 2004 and 2007, the proportion of households owning at least one ITN rose from 23% to 39%. During this period, the proportion of children under five years of age (U5s), and the proportion of pregnant women sleeping under an ITN increased from 16% to 26% and 16% to 27%, respectively [2,10,11]. However, the MoHSW and NMCP considered these increases in ITN ownership and use too low to reach RBM targets of 80% by 2010.

In 2007, following extensive stakeholder consultations, NMCP developed a plan in accordance with the current RBM strategy to rapidly increase ITN ownership and use through the procurement and delivery of a free long-lasting insecticidal nets (LLINs) to all U5s in mainland Tanzania [3,12]. This report summarizes the funding strategy for the national catch-up plan, as well as for the logistics and training to coordinate the timely and equitable delivery of LLINs at the village-level. It also presents the financial costs of this mass distribution and preliminary coverage data resulting therefrom.

## Methods

### Initiation and Financing of the U5 mass distribution campaign

In March 2007, the GFATM invited Tanzania's Country Coordinating Mechanism (CCM) to submit a Rolling Continuation Channel (RCC) application to extend its Round 1 grant to increase ITN ownership and use among vulnerable groups. After extensive stakeholder discussions, NMCP proposed the continuation of the voucher programme plus the launch of a free, one-time LLIN distribution campaign for U5s (under five catch-up campaign - U5CC). The World Bank under its Booster Programme for Malaria Control in Africa and PMI simultaneously contributed funds to expand the scope of the distribution. In addition, Malaria No More/UNICEF, World Vision Switzerland, the UK Department for International Development (DfID), and the Swiss Agency for Development and Cooperation (SDC) contributed additional funding. Contributions raised during the Davos Economic Forum in 2005 and unobligated MoHSW funds closed the final budgetary gaps to complete the national campaign.

### Tendering and procurement of LLINs and sub-contractors

In compliance with Tanzania's tendering rules and required product specifications, a single LLIN tender was issued by MoHSW funded by GFATM and the World Bank. LLINs financed by PMI were the subject of a separate tender. The only polyethylene net with the required qualifications at the time (2008), specifically a full WHO Pesticide Evaluation Scheme recommendation, was the Olyset^® ^net produced by Sumitomo Chemical and A-Z Textiles Ltd. This requirement was exceptionally agreed to by the three donors in order to ensure that the same LLIN was delivered throughout the country following initial delays negotiating this issue with the different donors lasting several months. Because the local manufacturer (A-Z Textiles) also won the contract for distribution to village level, the management of the logistics was greatly facilitated.

The five grant sub-recipients had already been identified through a competitive procurement mechanism conducted by the Country Coordinating Mechanism prior to the development of the RCC grant proposal. The MoHSW, through its Procurement Management Unit, contracted the grant sub-recipients for the five key components of the campaign: (1) Logistics - MEDA Economic Development Associates; (2) Training - World Vision Tanzania (WVT) (3) Social mobilization - Population Services International; (4) Monitoring and Evaluation - Ifakara Health Institute (IHI) who in turn sub-contracted technical support to the London School of Hygiene and Tropical Medicine; (5) Financial and procedural audit - KPMG. The proposal submission, evaluation and contracting process for the five sub-recipients took more than seven months to complete. The delivery contractor, (A to Z Textiles Ltd), was identified by the logistics contractor MEDA Economic Development Associates through a separate competitive tender. The LLIN Hang-up Campaign (conducted by Tanzania Red Cross) was separately contracted by USAID and co-funded by DfID.

### National coordination and regional stakeholder coordination

**Figure 1:**
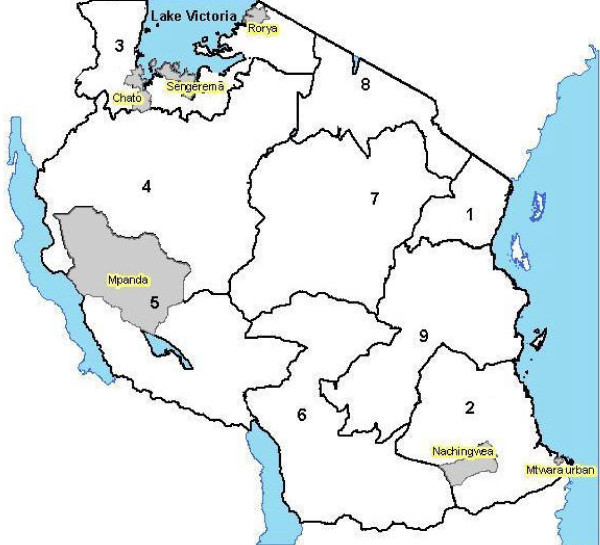
**United Republic of Tanzania, with Health Zones**. Highlighted districts indicates locations of household surveys (1 Tanga, 2 South, 3 Lake, 4 West Lake, 5 South West, 6 Southern Highlands, 7 Central, 8 North, 9 Coast)

**Figure 2:**
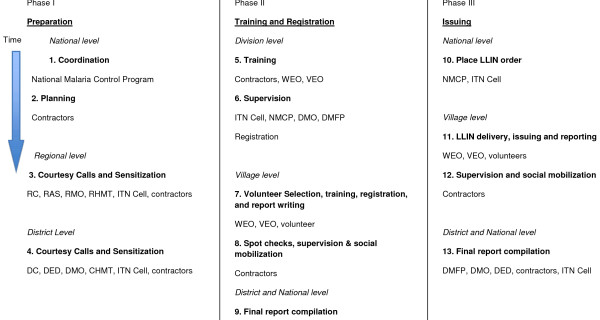
**Structure of the National, Regional, District and Local Government levels involved in the under-5 coverage campaign in Tanzania, 2008-09**.

Since 2003, the ITN Cell, a unit within the NMCP, has coordinated the National Insecticide-Treated Nets Program (NATNETS) programme, with technical and financial support from the Swiss Agency for Development and Cooperation through its executing agency, the Swiss Tropical and Public Health Institute. During the U5CC, the ITN Cell and other NMCP staff coordinated planning in Dar es Salaam and in the field where they facilitated contacts between the local government and the government contractors. The U5CC proceeded on a rolling basis, entering a new zone (comprising 2-3 regions each) every five weeks, while simultaneously completing later phases of the campaign in other zones. The U5CC covered Tanzania's eight zones in order of their malaria prevalence, with the zones having the highest malaria prevalence visited first. Tanzania's mainland population in 2009/10 exceeded 41 million, with over 80% living in areas with stable perennial to stable seasonal malaria transmission [4]. The U5CC started with a pilot programme in Mpanda District (highlighted in Figure 1) in October 2008 to test the planned methodology. The lessons from the pilot programme were incorporated into the main campaign, which is detailed below and diagrammed in Figure 2.

Prior to departure of the field team (NMCP, ITN Cell and contractors' staff) from Dar es Salaam, the MoHSW sent a letter to Regional and District government officials, alerting them to the upcoming U5CC in their respective regions and districts. Upon arrival in a region, the field team-consisting of contractors and an ITN Cell representative-jointly briefed the regional government authorities on the U5CC. The training contractor organized regional sensitization meetings for the regional authorities including the regional health management teams who had a role in regional supervision.

### District-level activities

#### Courtesy calls

At the district level, the field team engaged the District Medical Officer and the District Executive Director, briefed them on their U5CC related responsibilities and the need to invite local government officials, Ward and Village Executive Officers - WEOs and VEOs, to a training session on their oversight role in the U5CC. Any unfinished micro-planning was completed with the assistance of the district Malaria Focal Person. These Malaria Focal Persons are part of the district Council Health Management Teams (CHMT) and are responsible for coordinating all malaria-related activities in their district. The post was established in each of mainland Tanzania's 121 Districts in 2004 [13,14].

#### Sensitization and training

Following the introductory courtesy calls, the training contractor conducted sensitization meetings with the relevant district authorities, including the Council Health Management Teams, who played a critical role in supervision, report collection, and payments to volunteers engaged in the household registration and LLIN issuing processes. Immediately after the sensitization meeting, the training contractor conducted a two-day training session for WEOs and VEOs at each of the district's four to six divisions. WEOs and VEOs were trained on their responsibilities as direct supervisors of the village volunteers who conducted household registration and LLIN issuing processes. WEOs and VEOs received illustrated guides as a reference during supervision. VEOs were responsible for village volunteers' selection and training, oversight of LLIN storage and final distribution of the LLINs from the storage space to the village government posts where nets were issued during three campaign days (Friday-Sunday).

#### Household registration

Upon returning to their villages, VEOs selected four healthy, literate and respected members of the community to conduct a house-to-house registration. This five-day process involved recording the names of every U5 at each household in the village in the U5CC register book. The volunteers provided the child's care-givers with a sequentially numbered coupon (identifying the recipient's place in the register book) and instructions to bring the coupon to the LLIN issuing point on one of the designated issuing days. Upon the completion of registration, volunteers submitted their register books to the VEO who compiled all data into a village registration report. These village reports provided an estimate for the number of children eligible for an LLIN, including an estimate of the number of children missed during the registration, based upon the VEOs' data on U5s in the village. The WEO combined all village registration reports into a ward registration report, which the Council Health Management Teams collected while making payments for the registration process.

#### Community sensitization

Before and during each registration, the social mobilization contractor conducted promotional activities in the area to encourage residents to participate in registration. These activities included road shows, mobile video units (entertaining health-related documentaries displayed on large screens) public meetings, public address broadcasts from vehicles, radio discussions, radio advertisements, and print media.

### LLIN delivery, distribution, and issuing

#### Delivery and distribution

The LLIN needs for each zone were compiled into a village-level packing list and reviewed and approved by NMCP prior to order placement. In addition, the distribution contractor (also A-Z Textiles Ltd) was responsible for delivering (via truck) the appropriate number of LLINs to every village in a zone within 30 days of receipt of the order. These deliveries included enough LLINs to meet the village registration requirements as well as a 5% village buffer for unregistered children, an additional 5% bale rounding factor and (initially) a further 5% buffer at district level to compensate for any additional shortfalls in the villages. Upon reaching the village, A-Z Textiles delivered the LLINs only after the VEO provided a signature and official stamp to receive them, as the VEO was the custodian of the LLINs until the were issued. In preparation for issuing, each VEO then selected and trained two new volunteers on the LLIN issuing process and supervised the transfer of LLINs from their secure storage locations to the one or two issuing posts in each village.

#### Social mobilization

In the weeks preceding LLIN issuing, the social mobilization contractor returned to the regions with radio broadcasts, print materials, road shows, and mobile video units to prepare the public for the delivery of the nets.

#### LLIN issuing

Issuing was conducted over a weekend in order to ensure the maximum number of people was at home, yet always started on a Friday to reduce congestion from too many people attending on the first day. Caregivers of U5s were instructed to visit the issuing posts with their numbered coupons during any of the three LLIN issuing days. They were not required to bring their children provided they had been pre-registered. The caregivers presented their coupons to the volunteers, who verified the entry in the register book. Caregivers confirmed receipt of the nets, and the fingers of children (if present) were marked with indelible ink.

#### Unregistered children

On the final day of net issuing, volunteers began recording the names of children missed during the initial registration. These children received LLINs from the village or ward-level buffer stocks. Upon completion of issuing days, the VEO compiled a village-level report on the LLIN issuing and submitted it to the WEO, who prepared a ward-level report. These reports were collected by the Council Health Management Team members when they returned to the wards to make LLIN issuing payments. Unissued nets were distributed to any remaining unregistered U5s and then to vulnerable members of the community, including people living with HIV/AIDS, elderly, disabled, or very ill people, as identified by the village council.

### LLIN hang-up campaign

Approximately one month after LLIN issuance, an organized effort was made to encourage household members to hang-up and use their new LLINs. The effort was implemented by the Tanzania Red Cross volunteer network and local government officials (including WEOs and VEOs) in each zone. In areas lacking a Red Cross volunteer structure, division-level extension officers worked as supervisors and ward health workers served as volunteers. With one volunteer per village, each volunteer had 12 days to visit every household in the village to ensure the new LLIN was hung and used and to share malaria messages with residents by distributing illustrated leaflets. Volunteer reports were collected by local supervisors and entered into a national database.

### Monitoring and evaluation

The monitoring and evaluation contractor, Ifakara Health Institute, assisted by the London School of Hygiene and Tropical Medicine, conducted a series of post-distribution district household surveys on ITN coverage and other U5CC related activities one to three months following net issuing in a given zone. Districts were selected based upon the availability of baseline data from a 2008 nationally representative malaria survey, including five Districts, (Figure 1 - Ifakara Health Institute and the London School of Hygiene and Tropical Medicine 2009, unpublished data). From each district, 30 clusters (villages) were selected with probability proportional to the size of the village. Within each selected cluster, one sub-village was chosen randomly and 30 households were chosen from that sub-village, based upon a modified Expanded Immunization Programme sampling procedure [15]. Questions about household ownership and use were asked using the standardized Malaria Indicator Survey format

## Results

### Total campaign financial cost

Nine different donors funded the U5CC (Table 1), and contributions ranged from $25,258,382 (GFATM) to $317,817 (Davos World Economic Forum Fund). Table 1 represents the direct financial costs for the U5CC, not including local government contributions. All of these funds were provided in unadjusted USD, with the exception of the Swiss Agency for Development and Cooperation and MoHSW contributions, which were calculated in USD based upon the exchange rate at the time of money transfer. The primary donors (GFATM, World Bank, and PMI) committed funds in 2008. The overall cost of the U5CC totaled USD $63,831,113, of which USD $47,340,943 (74.2%) was used to purchase 9,034,677 LLINs. The financial cost per LLINs distributed was USD $7.07 per LLIN, of which USD $5.24 were used to purchased the net, and USD $1.83 were for LLIN transport, training, logistics, management, social mobilization/BCC, and M&E.

**Table 1:** Financial Costs (in un-adjusted United States Dollars) of an LLIN distribution by cost category and donor

Cost Category	Donor Contribution	Amount	Cost/LLIN* (% of cost)
LLIN ex-factory costs	Global Fund	19,172,566	
	World Bank†	12,681,600	
	PMI	9,679,570	
	Malaria No More/UNICEF	2,750,726	
	MoHSW	1,993,046	
	SDC	745,618	
	Davos (WEF)‡	317,817	
	Subtotal	47,340,943	$5.24 (74.2)

LLIN Delivery**	Global Fund	959,871	
	World Bank	1,401,425	
	PMI	1,195,653	
	MoHSW	171,996	
	MNM/UNICEF	226,133	
	SDC	36,752	
	Subtotal	3,991,830	$0.44 (6.2)

Logistics	Global Fund	2,029,306	
	World Bank	1,057,180	
	PMI	1,524,392	
	Subtotal	4,610,878	$0.51 (7.2)

Training	Global Fund	1,485,984	
	World Vision Switzerland	584,156	
	PMI	599,055	
	Subtotal	2,669,195	$0.30 (4.2)

Social Mobilization/BCC	Global Fund	1,189,837	
	PMI	1,500,000	
	Subtotal	2,689,837	$0.30 (4.2)

Hang-up Campaign	DFID	1,304,840	
	PMI	500,000	
	Subtotal	1,804,840	$0.20 (2.8)

Audit	Global Fund	150,853	$0.02 (0.3)

Monitoring & Evaluation	Global Fund	109,990	$0.01 (0.1)

Administration Costs	Global Fund	159,975	
	SDC	302,772	
	Subtotal	462,747	$0.05(0.7)

**Total**		**63,831,113**	$7.07(100)

*The cost category subtotal divided by the number of LLINs issued

† The World Bank also provided $10.17 million for a retreatment campaign that was to run alongside the LLIN distribution in order to re-treat existing nets.

‡Funding committed for malaria in Tanzania at the 2005 World Economic Forum

** Delivery costs refers to the costs of transporting LLINs from the factory to the village level

### LLIN procurement and delivery

Towards the end of the campaign, the U5CC faced a 1,822,954 LLIN shortfall due to the difference between the estimated number of LLINs needed for the U5s (original budget) and the actual numbers of LLINs ordered based upon the household registration process. The original budget relied upon National Bureau of Statistics (NBS) 2008 population projections from the 2002 Tanzania National Census, which projected a need for 7,220,083 LLINs. However, the actual order for LLINs in the first two zones (Southern and Lake) surpassed the original projections by 42%. An analysis detailed in Table 2 showed six main contributions to the discrepancy between the NBS estimates and actual LLIN needs. A total of 20.2% could be attributed to various buffers: 5% as village buffer, 5% at district level, 5% due to bale-rounding because only full bales of 40 LLINs were delivered to the village level, and a village level estimate of unregistered children capped at 5% of the total village LLIN order. A comparison between the NBS projections and 4,000 entries in a sample of U5CC register books from 12 villages indicated that another 7% of the discrepancy could be attributed to registration of overage children (>59 months), particularly five-year old children. The source of the remaining 14.8% discrepancy was most likely due to an NBS projection error or inaccurate census data in 2002. Table 3 illustrates the discrepancy between NBS estimates and LLIN orders on a zone basis. The total number of buffer LLINs provided for each zone was simply the difference between U5s registered and total LLINs sent.

**Table 2:** Components of the discrepancy between 2008 National Bureau of Statistics (NBS) estimates for children under five years and the actual LLIN order in Southern and Lake Zones

Source of Variance	Range (%)	Average (%)	Remaining variance after exclusion of each factor (%)
Buffer estimated by VEOs*	1.7-11.9	5	36.8
Additional 5% village buffer†	-	5	31.8
DMO 5% buffer‡	-	5	26.8
Additional nets due to bale rounding**	3-7	5	21.8
Over-registration of children aged five years††	0-20	7	14.8
Estimate of NBS projection error***	-	14.8	N/A

Total difference between order size and NBS projections	12-68	42	42

*Village Executive Officers (VEOs) estimate of the number of children under 5 years missed on the registration reports. This is capped at 5% of the total village order

†This automatic 5% buffer at the village level was removed following an analysis of the over-registration

‡This buffer was shifted to a 5% ward level buffer to better facilitate reallocation to village level

** This buffer was removed following an analysis of the over-registration

†† This estimation is derived from a comparison of age distribution between 4,000 register book entries and the NBS projections

*** This figure comes from the subtraction of all other factors

**Table 3:** Estimated and actual number of children under five years of age for determining LLIN needs by Zone, Tanzania, 2008-09

Zone	2008 NBS* Est. U5s from 2002 Census	U5s Registered	**LLINs delivered**†	**LLINS issued**‡ **(%)**	U5s not receiving an LLIN**
Tanga	288,185	N/A††	402,448	380,458(94.5)	0‡‡
South	333,571	403,594	469,644	435,112 (92.6)	3,456
Lake	1,457,439	1,925,372	2,107,000	2,047,889 (97.2)	65,618
West Lake	1,609,747	1,691,854	1,745,682	1,662,302 (95.2)	155,523
South West	782,563	766,291	793,696	789,586 (99.4)	68,859
Southern Highlands	464,119	514,414	562,112	554,433 (98.6)	22,922
Central	844,157	984,985	1,065,748	1,052,750 (98.7)	92,397
Northern	476,131	540,785	586,720	567,348 (96.6)	43,190
Coast	966,171	1,153,715	1,311,987	1,263,560 (96.3)	N/A
**Total**	**7,222,083**	**7.981,010**	**9,045,037**	**8,753,438 (96.7)**	**451,965**

*National Bureau of Statistics estimates based on 2008 population projections from the 2002 Census data

† LLINs delivered by delivery contractor to the village

‡ LLINs issued to U5s during campaign days

** Refers to unregistered children who came to distribution point on one of the three distribution days, but did not receive an LLIN

†† This zone was implemented with a slightly different methodology, due to a pilot 'integrated distribution'

‡‡ Following the distribution, additional LLINs were procured and delivered to village level to meet the shortfall

### LLIN distribution and issuing

Following the Table 2 analysis (conducted after LLIN issuing in the first two zones), policies on buffer stocks became more restrictive to reduce the anticipated future shortfall of nets. Overall, the vast majority (96.1%) of children that attended issuing posts received LLINs, as illustrated in Table 3. This included the children registered by volunteers as well as the children missed in the registration process who received LLINs from buffer stocks. Village reports documented that a total of 222,712 LLINs (the difference between LLINs delivered to a village and LLINs issued to U5s) were delivered to villages in excess of the village's U5 registered population. These LLINs were reallocated to 1) unregistered U5s in the village; 2) unregistered U5s in the ward; and (if any surplus LLINs remained) to needy members of the community. While the village and ward authorities were tasked with reallocating the LLIN surpluses, this occurred after the reporting period ended, so there is no data on this re-allocation.

Table 4 details the line item costs associated with the U5CC implementation in one of mainland Tanzania's 121 districts. The U5CC budget for Rural Kyela District was selected as an illustrative example of components of U5CC district budgets. This district is smaller in U5 population (38,007) than the average Tanzanian district (68,444), but the contractor budgets were comparable to other Tanzanian districts [16]. These costs exclude the LLIN procurement and delivery to village.

**Table 4:** Actual U5CC costs by activity, Kyela District, Tanzania

	No. people	No. units	Rate ($)	Cost ($)	Purpose
**TRAINING**					

**Divisional secretaries, WEOs and VEOs for registration and distribution**					

Subsistence & Travel	2	122	22.22	$5,422	2 DS, 15 WEOs, 105 VEOs

Venue	1	5	37.04	185	Training

District focal person	1	14	14.81	207	Allowance

Subtotal				5,814	

**Facilitation costs**					

Trainers	2	21	38.52	1,618	Subsistence

Photocopies	1	522	0.07	39	For training materials

Trainer Facilitation	2	1	118.4	236.8	Internet access/photocopies

Travel costs	15	1	7.41	111	District officials

Venue	1	1	779.3	779.26	Meeting, refreshments, and stationery

Subtotal				2,784.1	

Total Training				8,599	

**LOGISTICS**					

**General fees**					

Coordinator	2	10	37.04	741	For DMO & CHMT staff facilitation

Contingency	4	4	74.07	1,185	In the event of emergencies

Police escort	8	4	7.41	237	Two police escorts to bank

CHMT escort	4	4	14.81	237	CHMT escort to bank

Subtotal				2,400	

**Supervision/payments for registration and issuing (4 routes)**					

Supervisor day	3	14	14.81	$622	CHMT to do supervision, payments

Supervisor night	3	12	22.22	800	CHMT to do supervision, payments

Driver day	3	14	7.41	311	Driver to accompany CHMT member

Driver night	3	12	18.52	667	Driver to accompany CHMT member

Fuel	3	580	1.26	2,191	District vehicles

Boat hire	2	2	37.04	148	Boat hire for supervision

Police escort	4	9	29.63	978	Two police escorts on the route

WEO	10	8	14.81	1,185	Lump sum to each WEO

VEO	54	8	22.22	9,600	Lump sum to each VEO

Village Chairperson	54	8	22.22	9,600	Lump sum to each VCP

LLIN transport fee	54	8	7.41	3,200	VEO to ensure LLINs reach issuing points

Registration volunteers	216	8	22.22	38,400	Four volunteers per village

Issuing volunteers	432	1	22.22	9,600	Two volunteers per issuing point

Emergency volunteers	16	3	7.41	356	For larger-than-expected issuing posts

Contingencies				348	In the event of emergencies

Subtotal				78,006	

**Special supervision of issuing only**					

Subtotal	3	1	91.75	275	Fuel and per diem DC, DED, and DMO

Total Logistics				80,681	

**SOCIAL MOBILIZATION**					

PSI Staff	10	10	135.3	1,353	Subsistence Hotel/per diem

Mass media		1	485	485	Radio spots/Live talks

Road shows	15	15	306.6	4,599	Hiring trucks, entertainers

T-shirts		40	3.4	135	Promotional materials

Print materials		141,036	0.0005	71	Social Mobilization materials

Mobile Video Unit		10	15.9	159	Mobile theaters with malaria messages

Fuel	10	10	72	720	For the social mobilization activities

Total Social Mobilization				7,522	

**Grand Total**				**$97,783**	

### Hang-up campaign

#### Net coverage following campaign

The map in Figure 1 indicates the districts selected for post-U5CC surveys (Nachingwea, Mtwara Urban, Sengerema, Chato, and Rorya) as well as the Mpanda pilot district. Household ITN ownership increased from a national average of 45.7% in 2008 to 63.4% in 2009 (ranging from 60.8% in the South Zone to 82.0% in the Lake Zone). Children under five years (U5s) ITN coverage following the Roll Back Malaria definition means the percentage of U5s sleeping under an ITN the night before the survey. It increased nationally from 28.8% in 2008 to 64.1% in 2009, a 2.2-fold increase. Increases ranged from 25.9% to 48.0% in the Southern Zone and 23.9% to 62.2% in the Lake Zone. The Tanzania Red Cross reported that volunteers visited households with a total of 9,080,232 U5s and assisted in hanging 1,702,840 LLINs.

The 2009-2010 Demographic Health Survey took place following the U5CC and Hang-up Campaign in 14 regions, at the same time of the campaign in five regions, and before the campaigns in the remaining three regions. These additional ITN coverage data are presented in the Discussion.

## Discussion

The combination of funding sources was in itself an achievement, demonstrating the attractiveness of the U5CC to donors and hence the willingness of numerous agencies to collaborate [17]. However, achieving agreement among the main donors on LLIN tender specifications represented a particular challenge. Initially the MoHSW expressed its preference for a more durable (but also more expensive) polyethylene net, but GFATM, PMI and World Bank's procurement regulations precluded this without a clear and objective justification. After negotiations, MoHSW and these three donors reached a consensus that met the requirements for Tanzania's LLIN policy.

At USD $7.07 (unadjusted) per LLIN, Tanzania's distribution compares favorably with other recent LLIN deliveries, including distributions in Eritrea, Malawi, Senegal, and Togo, as well as Tanzania's ongoing LLIN voucher plan for pregnant women and infants. The free or partially subsidized distribution costs in these countries ranged from USD $6.90 to $9.50 and their mechanisms included both public and mixed public private sector distributions [18,19].

The U5CC used local government employees rather than health workers to avoid further burdening the Tanzanian healthcare system. This was a major strength of the campaign because 66% of Tanzania's healthcare staff positions are unfilled [20]. As civil servants, these local government officials were not affiliated with any political party and most had offices where the LLINs could be securely stored until the issuing was completed. In addition, these officials reported directly to their District Executive Directors, who had already expressed support for the successful completion of the U5CC. Further, the U5CC engaged community leadership to help increase accountability and transparency.

The U5CC roll-out in a given zone occurred in a 3-month cycle, with one month for training and sensitization, one month for registration, report writing and LLIN order placement, and one month for LLIN delivery, issuing, and report writing. The registration process provided village-level details of LLIN needs and intended recipients, an unprecedented level of detail and transparency. In addition, A-Z Textiles required 30 days to deliver the exact number of LLINs requested to each village in the zone. This contrasts with the delivery mechanisms in Zambia in 2003, which did not have a registration and where local authorities were responsible for moving the ITNs to village level [21]. The longer time-frames of the U5CC roll-out were a result of both the accuracy of the LLIN distribution and the scale of the LLIN distribution in each zone, where over a million LLINs were delivered.

Every five weeks, the training and sensitization team entered a new zone to begin training, as registration continued in the previous zone and LLIN issuing continued in the zone before that. Because each zone contained two to three regions, the respective capacities of the contractors could not accommodate additional regions or zones in the month-by-month roll-out. While this necessitated a total of twelve months before U5CC completion, it also enabled the coordinators to closely monitor the roll-out in each region and to address problems or shortfalls. For distributions covering a several district or small countries such as Togo, all of the U5 LLINs were distributed over a short period as part of an integrated campaign [22,23]. Because of Tanzania's size, each of the eight zone LLIN distributions was approximately the size of Togo's entire national LLIN distribution campaign [23].

Following the receipt of the registration data and LLIN order placement in the first two zones, it was apparent that LLINs distributed according to the registration figures plus the village (5%), bale rounding (5%) and district level (5%) buffers would lead to a significant funding shortfall and there would be insufficient nets for the whole country. These buffers had been introduced following the widespread LLIN shortfalls in the Mpanda District pilot distribution when the LLIN order exactly matched the house-to-house registration data. As a result, NMCP assessed the discrepancies between the original NBS data and the actual LLIN needs for each region in the first two zones. It was decided that the district buffer and the additional bale-rounding buffer could be eliminated to conserve LLINs for the later stages of the campaign.

Despite the elimination of two buffers, the U5CC still faced an additional USD $10 million shortfall to distribute LLINs in all zones. The U5CC coordinators solicited funds from donors to close this gap. The final contribution of $1,993,046 from the Tanzanian government supported the purchase of LLINs for the three districts of Dar es Salaam, which allowed the U5CC to draw to a close. As a result of the buffer reductions, 451,965 U5s who had not registered for the U5CC, but had gone to issuing points to receive an LLIN left without one. These children will receive free LLINs in the upcoming universal coverage campaign, supported by GFATM Round 8.

**Figure 3:**
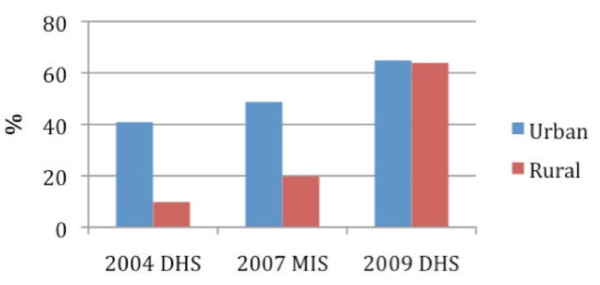
**A comparison of U5 ITN coverage between urban and rural populations from three nationally representative surveys**.

Preliminary data in the first two zones showed marked improvement in two of the RBM core indicators: ITN ownership and U5 ITN use. In both zones, U5 ITN use lagged behind ownership by 12-20 percentage points. This is consistent with other mass LLIN distribution campaigns where percentage point gaps of 36.0 and 36.1 between ownership and use were noted in Kenyan household members and Ghanaian U5s, respectively [24,25]. An assessment of LLIN needs for sub-Saharan Africa have assumed 55% ITN use with 80% ITN ownership [26]. Additional behavioural change communication to address the importance of sleeping under an ITN every night will likely assist in closing this gap [27]. The NMCP launched a "focal parents programme" and a "community change agent programme" to assist in improving ITN use at the community level through the training of key community members on malaria prevention and treatment. Even before these behavioural change efforts reach full scale-up, Tanzania has experienced improvements as described in the preliminary report of the 2009-10 DHS [11]. ITN use among children under five years and pregnant women are currently at similar levels as household ownership of at least one ITN (Table 5.) Figure 3 indicates that equity in U5 ITN coverage improved significantly following the U5CC.

**Table 5:** Household ITN ownership & ITN use among children under five years of age determined through household surveys, Tanzania, 2008 (pre-campaign) - 2009 (post-campaign)

	Household ITN ownership				U5 ITN use*			
**Area**	**2008**		**2009**		**2008**		**2009**	

	**N**	**% Ownership (95% CI)**	**N**	**% Ownership (95% CI)**	**N**	**ITN use Children <5 (95% CI)**	**N**	**ITN use Children <5 (95% CI)**

Southern Zone	875	46.3 (39.7-52.9)	592	60.8 (56.8-64.6)	638	25.9 (21.0-31.4)	304	48.0 (42.3-53.8)

Lake Zone	1176	34.1 (28.2-40.5)	891	82.0 (79.4-84.5)	1408	23.9 (19.1-29.4)	1184	62.2 (58.8-65.5)

Tanzania	7200	45.7 (40.1-51.5)	NA	63.4†	5701	28.8 (22.3-36.3)	NA	64.1†

*Percentage who slept under an ITN the past night; an ITN is a 1) factory treated net that does not require any further treatment, or 2) a pre-treated net obtained in the past 12 months, or 3) a net that has been soaked with insecticide within the past 12 months.

† Preliminary data from 2009-2010 Tanzania Demographic Health Survey

The U5CC will be immediately followed by a universal coverage campaign funded by GFATM Round 8. The goal of this additional campaign is to provide 18 million LLINs for every sleeping space not covered by an U5CC LLIN. Beyond these free distribution (catch-up) campaigns, future free LLIN distributions remain uncertain. However, a regular mechanism of sustaining the current high ITN coverage through continuous distribution mechanisms is clearly needed. Tanzania has developed and sustained an ITN voucher programme for pregnant women and infants since 2004 [6,7,16]. This successful voucher programme was recently upgraded by the Global Fund RCC and PMI to provide an LLIN with a fixed co-payment of TSH 500 (approximately US $0.35) per voucher redemption. If funding continues, this mechanism will serve as one part of the national keep-up strategy until a new policy for ITN distribution is developed. Additional mechanisms for ITN distribution will be needed to ensure a sufficient, continuous flow of replacement ITNs to the Tanzanian population to sustain high ITN coverage rates. This is a situation common to all countries that have completed campaigns, and more discussions at both national and global level are urgently required to explore the best possible options for the future.

## List of abbreviations

CHMT: Council Health Management Team; DED: District Executive Director; DfID: Department for International Development; DHS: Demographic Health Survey; GFATM: Global Fund to Fight AIDS TB and Malaria; IHI: Ifakara Health Institute; ITN: Insecticide-treated net; LLIN: Long-lasting insecticidal net; LSHTM: London School of Tropical Medicine and Hygiene; MEDA: MEDA Economic Development Associates; MFP: Malaria Focal Person; MoHSW: Ministry of Health and Social Welfare; NATNETS: National Insecticide-Treated Nets Program; NBS: National Bureau of Statistics; NMCP: National Malaria Control Program; PMI: Presidents Malaria Initiative; RBM: Roll Back Malaria; RCC: Rolling Continuation Channel; RHMT: Regional Health Management Team; SDC: Swiss Agency for Development and Cooperation; Swiss TPH: Swiss Tropical and Public Health Institute; TNVS: Tanzania National Voucher Scheme; TRC: Tanzania Red Cross; U5: Child under five years of age; U5CC: Under 5 Catch-up Campaign; USD: Unites States Dollars; VEO: Village Executive Officer; WEF: World Economic Forum; WEO: Ward Executive Officer; WVT: World Vision Tanzania;

## Competing interests

The authors declare that they have no competing interests.

## Authors' contributions

AM, CL & NJ designed the methodology and JN, RM, SO & AM made substantial contributions to the implementation and the analysis of the methodology. RN provided data on the results of the methodology and provided substantial contributions to the manuscript. NK participated in methodology implementation and coordination of the manuscript development and approval. PM made substantial contributions to the design of the methodology and extensive contributions to the development, drafting, analysis, and editing of the manuscript. KB participated in the implementation of the methodology and drafted the manuscript. All authors read and approved the final manuscript.
